# Comprehensive human locomotion and electromyography dataset: Gait120

**DOI:** 10.1038/s41597-025-05391-0

**Published:** 2025-06-18

**Authors:** Junyo Boo, Dongwook Seo, Minseung Kim, Seungbum Koo

**Affiliations:** Department of Mechanical Engineering, Korea Advanced Institute of Science and Technology, Daejeon, Republic of Korea

**Keywords:** Health care, Biomedical engineering

## Abstract

Understanding human locomotion patterns and their variations requires comprehensive data across different age groups and movement tasks, given the complexity of the human musculoskeletal system. This study presents a dataset of human locomotion during daily activities, collected from 120 healthy male participants (age range: 20–59 years). The experimental protocol included seven distinct tasks: level walking, stair ascent/descent, slope walking (ascent/descent), and sit-to-stand/stand-to-sit movements. Data were collected using an optical motion capture system, force plates, and surface electromyography sensors on the right lower limb. The final dataset includes 6,882 movement cycles across all tasks, including full-body joint kinematics and muscle activity patterns. This comprehensive dataset will contribute to understanding the variations in movement patterns and muscle activation during common daily activities across a broad adult male population.

## Background & Summary

Joint kinematics result from a complex combination of neural control, neurological reflexes, muscle coordination, connective tissue stresses, and joint mechanics^1^. Combined with electromyography (EMG), joint kinematics provides valuable information for investigating the effects of abnormalities in individual and combined factors^2^. When integrated with force plate data, joint kinematics enables understanding of internal body forces, including muscle and ligament tensions and joint contact forces during locomotion^3^.

Joint kinematics for locomotion studies is typically obtained using optical motion capture systems and reflective body markers^4,5^. Body markers in a human model are aligned with the optical markers from the motion capture system to obtain joint angles through inverse kinematics algorithms. Human motion processing software such as OpenSim^6,7^ and AnyBody^8^ (AnyBody Technology, Aalborg, Denmark) provides specific human models and performs inverse kinematics to generate temporal joint kinematics. Software with musculoskeletal models can perform inverse dynamics using joint kinematics, force plate, and EMG data to calculate internal muscle forces along with joint moments. These procedures facilitate data acquisition of joint kinematics and muscle dynamics, essential for predicting musculoskeletal injuries and analyzing movement characteristics in patients with musculoskeletal disorders^9,10^.

Furthermore, motion capture data serve as reference movements in deep learning applications for virtual locomotion simulations^11^. These data have been applied in musculoskeletal model reinforcement learning, demonstrating utility across various fields, including exoskeleton studies^11,12^, metabolic energy consumption prediction^13^, and prosthetics development^14^. These applications highlight the data’s versatility and potential impact across healthcare and technology domains.

Surface EMG (sEMG) data provide crucial insights into muscle activity and interactions not visible through motion capture alone. Integrating sEMG data into simulations enables comprehensive understanding of neuromuscular control during movement^15,16^. This integration is vital for designing effective rehabilitation protocols, enhancing sports performance, and developing advanced prosthetic control systems.

Clinical gait studies focus on analyzing pathological movement patterns^17–19^, comparing collected data with healthy individuals’ movements. Surgical and non-surgical interventions on the lower limbs aim to restore normal function of the joints. Therefore, it is essential to understand gait variations in healthy individuals and the effects of physical characteristics such as weight, limb length, and gait speed^20^.

Among the cyclic movements performed in daily life, actions that vary with the environment include walking and sit-to-stand transitions. Traditional methods for measuring cyclic movements utilize optical motion capture systems in laboratory environments^21–24^. To reduce the burden of controlled environment setups, wearable sensors such as Inertial Measurement Units (IMUs) have been employed to capture movements in natural, everyday settings^25–27^. While IMU-based measurements offer flexibility in data acquisition, previous studies have reported slight differences compared to optical motion capture systems, attributed primarily to methodological differences between the sensor types rather than measurement inaccuracies^28–30^. Furthermore, data collection using wearable sensors in outdoor or natural environments often requires extended durations and faces challenges in maintaining consistent experimental conditions.

Numerous publicly available datasets provide EMG or gait data separately; however, our dataset uniquely combines synchronized full-body kinematic and EMG data from various cyclic tasks (level walking, stairs, slope, sit-to-stand, stand-to-sit). The controlled laboratory environment utilized in our study ensures high consistency, reproducibility, and accuracy, distinguishing our dataset from typical everyday-life datasets acquired via wearable sensors.

This dataset has been created to serve as a comprehensive locomotion reference across a broad age range of healthy individuals, with the goal of supporting various research applications in biomechanics and movement science. We analyzed and organized full-body motion capture data and lower limb muscle sEMG collected from 120 healthy male participants performing predefined daily movements (level walking, stair walking, slope walking, and sit-to-stand/stand-to-sit movements). All participants were free from musculoskeletal or neurological disorders. The analysis addressed kinematic and kinetic data, and muscle activity levels during movement performance. This study details participant group characteristics, data collection procedures, equipment specifications, experimental protocols, and data processing and validation methods. The final validated dataset includes 6,882 movement (or gait) cycles from seven different tasks, including level walking (1,060 gait cycles), stair ascent (1,187 gait cycles), stair descent (1,186 gait cycles), slope ascent (1,172 gait cycles), slope descent (1,150 gait cycles), sit-to-stand (558 movement cycles), and stand-to-sit (569 movement cycles). For each cycle, comprehensive full-body joint kinematics and muscle activity patterns are provided. The dataset is expected to facilitate investigations into variations in movement patterns and muscle activation during common daily activities among adult male populations.

Several publicly available datasets have supported human locomotion research by providing kinematic, kinetic, and EMG data. One dataset includes full-body motion capture, ground reaction forces, and EMG signals from 50 healthy adults walking at five controlled treadmill speeds^31^. Another dataset offers kinematic, kinetic, and EMG recordings from 50 participants aged 6 to 72 years, performing various locomotor tasks such as level walking, toe- and heel-walking, and stair ambulation^32^. A third dataset features lower-limb biomechanics and wearable sensor measurements, including IMUs and EMG signals, collected from 22 healthy individuals under diverse locomotion modes and terrain conditions^33^. Additional dataset provides comprehensive data on sit-to-walk transitions, including lower body motion capture, ground reaction forces, surface EMG, and IMU data from 65 healthy adults across various age groups^34^. More recently, a dataset has been released that focuses on above-knee amputees, capturing kinematic, kinetic, and EMG data during sit-to-stand and stand-to-sit transitions^35^.

Building on the strengths and addressing the limitations of these existing datasets, the present dataset offers several enhanced features. It provides synchronized full-body kinematic and surface EMG data from 120 healthy adult males performing seven distinct daily tasks, including level, stair, and slope walking, as well as sit-to-stand and stand-to-sit transitions. All data were collected in a controlled laboratory setting using optical motion capture and wireless EMG systems. This combination of high participant number, task variety, and sensor synchronization distinguishes the dataset as a valuable resource for biomechanical, clinical, and machine learning applications.

## Methods

### Participants

One hundred twenty male participants without musculoskeletal disorders participated in the experiment (age: 35.4 ± 12.8 years, height: 173.2 ± 5.6 cm, weight: 74.1 ± 12.5 kg; mean ± standard deviation [SD]). None of the participants exhibited any skin allergies to the alcohol swabs and fixation tape. This study was approved by the Institutional Review Board (IRB No. KH2023-053) at Korea Advanced Institute of Science and Technology (KAIST) to conduct the study and publicly share the anonymized data. Written informed consent was obtained from all participants prior to their participation in the experiment.

### Experimental environment

**Fig. 1:**
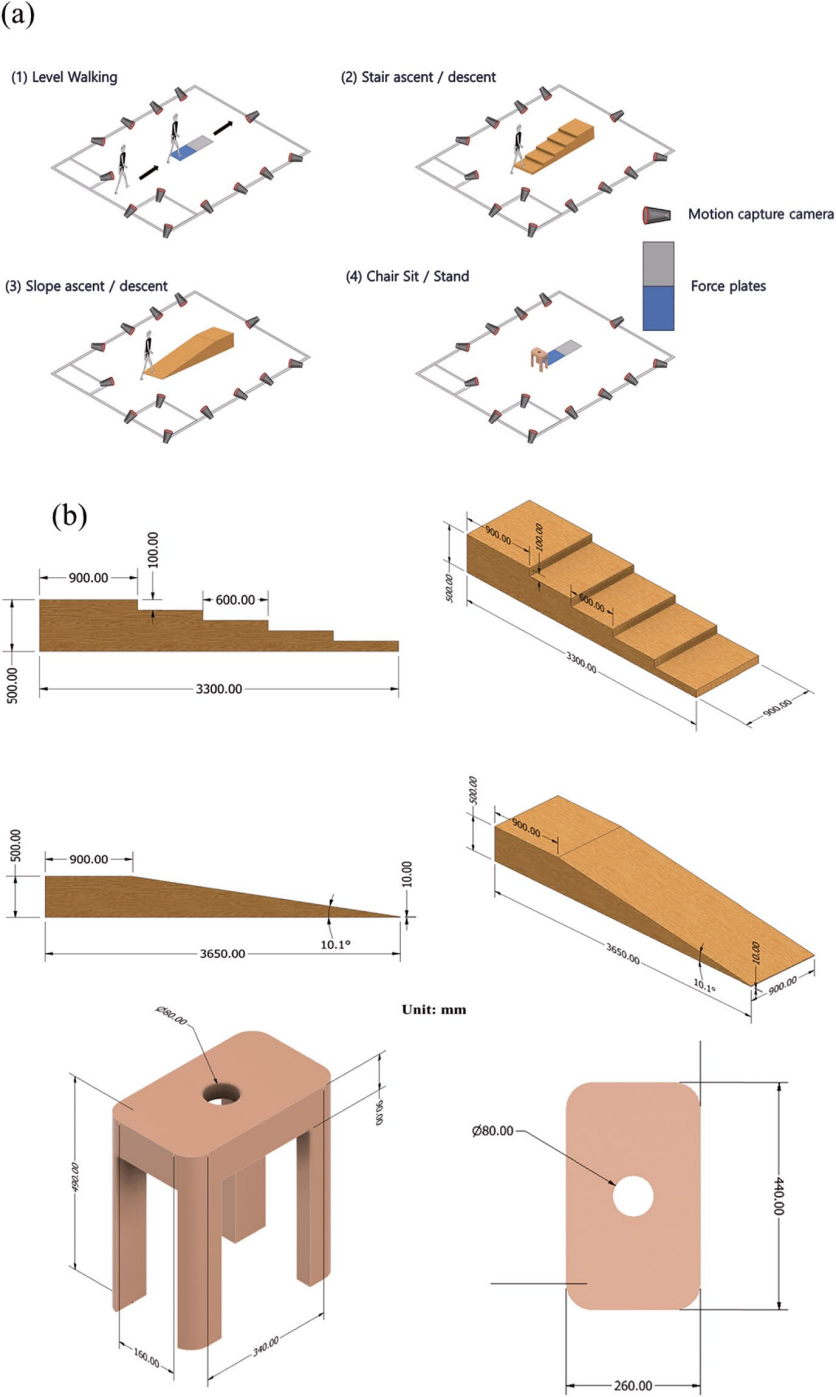
Experimental setup showing the motion capture environment and equipment configuration (**a**), and detailed specifications of the custom-designed stairs and slope (**b**).

All data were collected at a motion laboratory at KAIST, which is equipped with optical and digital devices for precise kinematic and kinetic data acquisition. Additionally, the laboratory features various locomotion test setups, including stairs, slopes, and stools, to facilitate a wide range of locomotion scenarios. The experimental environment is described in Fig. 1(a).

Specially designed stairs and a slope, created to mimic movements encountered in daily life, were used for the experiments. The stairs and slope were constructed as modular units for easy assembly and disassembly. Detailed structures of the stairs, slope, and stool are shown in Fig. 1(b). The height of the stool was 490 mm.

### Motion capture and electromyography details

The experimental sessions were conducted with 13 Vicon infrared cameras (T160, T40, T40S, Vantage; Vicon Ltd., UK) and two force plates (BMS400600; AMTI, USA; size: 400 mm × 600 mm each). The T160, T40, and T40S cameras were connected to a Vicon MX Giganet (camera data synchronization device), while the Vantage cameras were connected via a network switch (DGS-1520-28MP; D-link Inc., Taiwan). Data from motion capture cameras, force plates, and sEMG sensors (Avanti; Delsys Inc., USA) were synchronized and recorded concurrently using Vicon Nexus software (v2.10; Vicon Ltd., UK). Marker trajectory, ground reaction force (GRF), and sEMG data were simultaneously captured at sampling rates of 100 Hz, 1000 Hz, and 2000 Hz, respectively. The force plates were flush-mounted to match the height of the surrounding access floor and securely anchored to the underlying concrete building floor, ensuring that participants experienced no height changes or uneven surfaces during walking trials. GRFs were recorded only for level walking, sit-to-stand, and stand-to-sit tasks.

### Experimental preparation

All participants were thoroughly briefed on the experimental procedure and signed informed consent forms, including consent for data publication. They were provided with properly fitted clothing, including tight-fitting shorts and T-shirts, selected according to their body size. Body parameters were measured, including height, weight, leg length, knee and ankle width, shoulder offset, elbow and wrist width, and hand thickness.

Twelve wireless sEMG sensors were attached to different muscles of the right leg: vastus lateralis (VL), rectus femoris (RF), vastus medialis (VM), tibialis anterior (TA), biceps femoris (BF), semitendinosus (ST), gastrocnemius medialis (GM), gastrocnemius lateralis (GL), soleus medialis (SM), soleus lateralis (SL), peroneus longus (PL), and peroneus brevis (PB). Prior to sensor attachment, skin contaminants and excessive body hair were removed using alcohol swabs and disposable razors. The sEMG sensors were positioned according to the SENIAM guidelines^36^.

Maximum voluntary contraction (MVC) measurements were systematically conducted in five distinct stages, each adopting standardized postures and contraction methods referenced from previous studies^37–40^. (1) VL, RF, and VM: Participants were seated with their knees flexed approximately 90°, performing maximal knee extension against manual resistance applied at the ankle, sustained for five seconds. (2) TA: Participants remained seated and performed maximal dorsiflexion against manual resistance applied to the dorsum of the foot, maintaining the contraction for five seconds. (3) BF and ST: Participants lay prone with knees flexed approximately 45°, executing maximal knee flexion against manual resistance at the ankle, sustained for five seconds. 4) GM and GL: Participants performed maximal heel raises while standing, with external resistance applied at the shoulders, maintaining the contraction for five seconds. (5) SM, SL, PL, and PB: Participants sat with a resistance band looped over their forefoot, performing maximal plantarflexion and eversion movements, maintaining each contraction for five seconds.

**Fig. 2:**
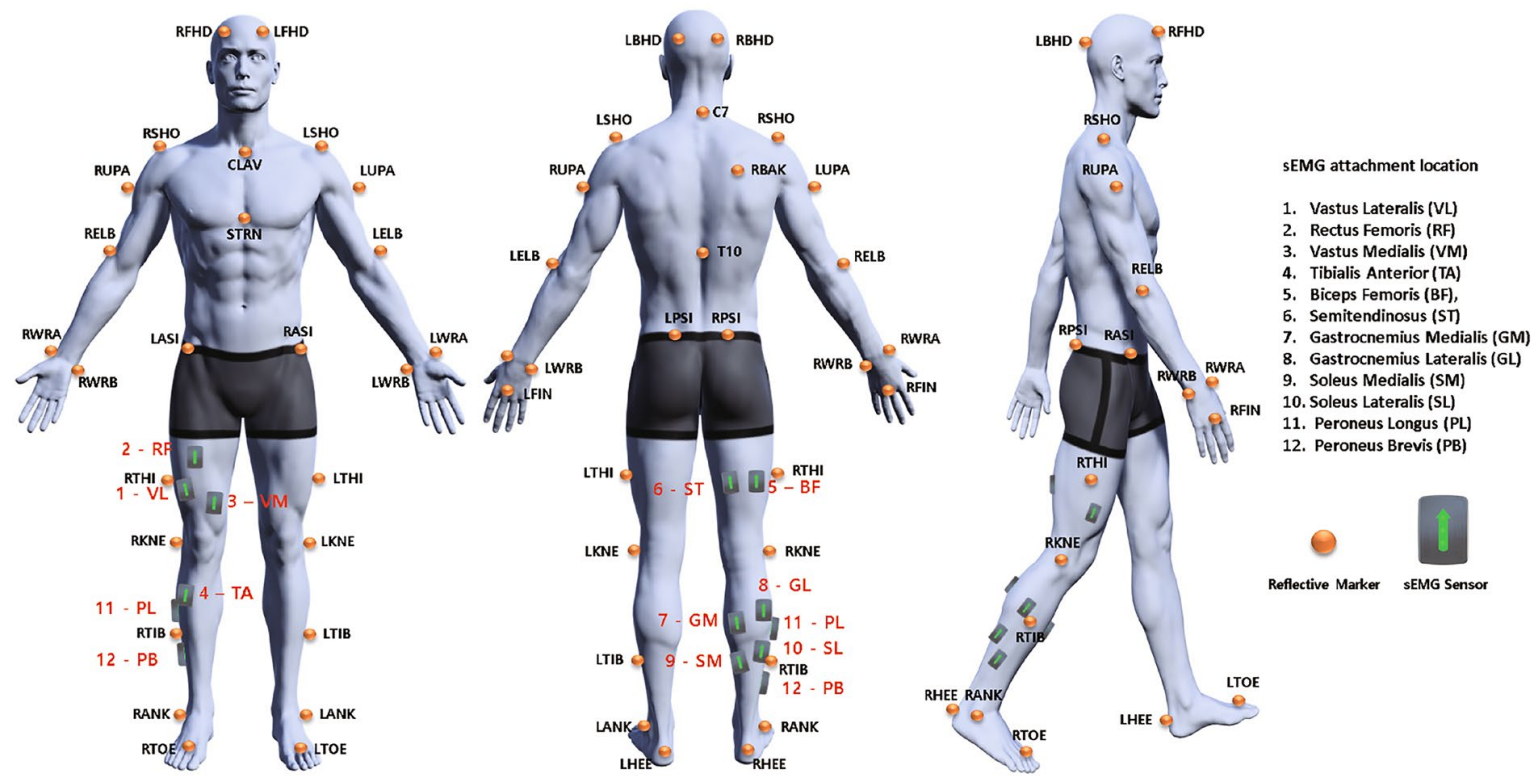
Marker and sEMG placement diagram showing the configuration according to the Plug-in-Gait model, with specific focus on lower limb sensor placement for muscle activity and joint kinematic measurements. Human model images were created with Magic Poser (Wombat Studio, Inc.) with permission.

Thirty-nine reflective markers (14-mm diameter) were attached following the Plug-in-Gait model marker set guidelines^41^ (Fig. 2). Marker positions and sEMG data quality were verified during a static pose held for at least ten seconds.

### Experimental procedure

Participants performed a warm-up and walked barefoot to acclimate to the equipment until they felt comfortable. The experiment consisted of seven distinct tasks, performed at self-selected speeds: (1) Level walking, (2) Stair ascent, (3) Stair descent, (4) Slope ascent, (5) Slope descent, (6) Stool sit-to-stand, and (7) Stool stand-to-sit. Participants completed a minimum of five trials per task. A five-minute rest period was provided between tasks, during which the researchers reconfigured the experimental setup (stairs, slope, or stool) as needed. During the Level walking task, participants could visually identify the force plates. Multiple preliminary walking trials were conducted, during which each participant’s starting position was individually adjusted to ensure natural and consistent foot placement on the force plates. Trials in which participants missed the force plates or stepped on their boundaries were discarded, and additional trials were performed to obtain valid data.

### Data processing

Motion capture marker data were labeled and gap-filled using Vicon Nexus software (v2.10; Vicon, Oxford, UK), utilizing spline and pattern filling with a maximum gap of 20 frames. The processed data were exported in multiple formats: marker trajectory data as .trc files, and force plate data as.mot files. Marker trajectory data were filtered using a Woltring filter^42^. The Woltring filter automatically determines the optimal smoothing parameter via generalized cross-validation, removing subjective bias in selecting cut-off frequencies and ensuring consistent, objective smoothing across all tasks.

For level walking, heel-strike and toe-off events of the right foot were identified from the force plate data. Heel-strike was defined as the moment when the vertical ground reaction force first exceeded 20 Newtons, and toe-off as the instant when it dropped below 20 Newtons. For stair and slope tasks, these events were manually specified using foot marker trajectories. For stool tasks, the first and last frames were identified using pelvis and shoulder marker trajectories^43^. For all locomotion tasks, at least one complete step cycle (right foot heel-strike to next right foot heel-strike) was extracted per trial, with two steps extracted when possible. For stool tasks, a single sit-to-stand or stand-to-sit motion was extracted per trial. Full-body joint angles were calculated using the inverse kinematics tool in the OpenSim^6^ software (v4.0). The analysis used a modified Rajagopal model^44^, with only one rotational degree of freedom in the knee joint. The coordinate system followed the OpenSim^6^ standard, with the X-axis pointing anteriorly (forward), Y-axis superiorly (upward), and Z-axis laterally (to the right). Joint angles were calculated using the OpenSim’s default Euler angle convention, with a rotation sequence of Z (flexion/extension), X (adduction/abduction), and Y (internal/external rotation). Joint angles were expressed in degrees.

Raw sEMG data from MVC and experimental tasks were exported as .csv files from Vicon Nexus and converted to.mat format using MATLAB R2023b (MathWorks Inc., Natick, MA). The EMG processing pipeline included: mean centering, Butterworth band-pass filtering (20–500 Hz), absolute value computation, Hampel filtering (window size: 250, sigma threshold: 3) with median replacement for outliers, and signal rectification using windowed root mean square (RMS) filter (window size: 250). Both motion capture and sEMG data contained synchronized frame index information for each trial. Therefore, the processed sEMG data were segmented based on frame indices identified from the corresponding motion capture data.

MVCs for each muscle were calculated using both the processed MVC sEMG signal and task-specific sEMG signals, defined as the maximum value across both signals^45^. The segmented sEMG data were then normalized to a 0–1 range by dividing the processed signal data values by calculated MVCs. The normalized sEMG data were interpolated to have uniform length for single steps using the cubic b-spline method, with first and last frames set to 0 and 1, respectively.

## Data Records

**Fig. 3:**
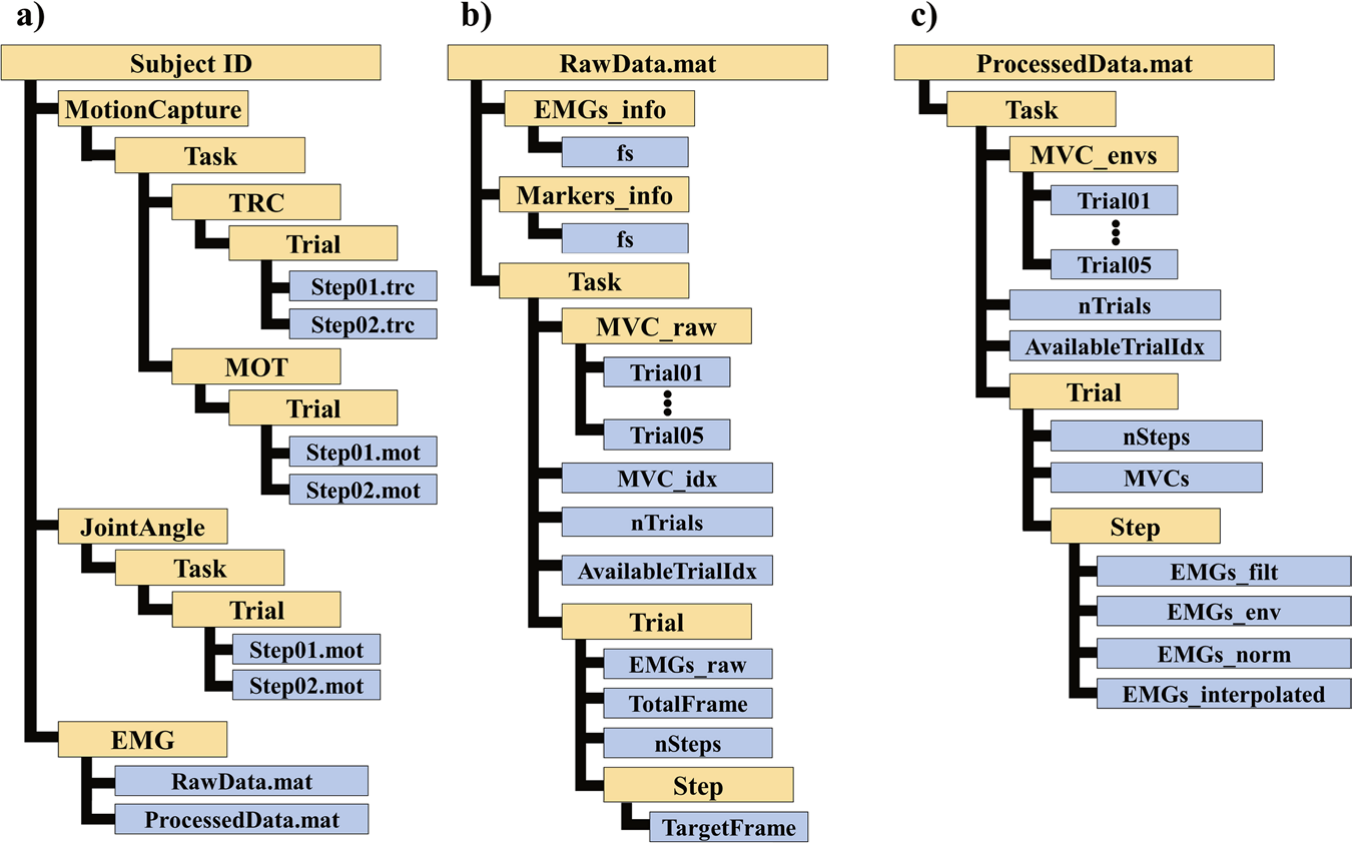
Data Organization and File Structure. (**a**) Visual representation of the folder structure showing the organization of EMG data, motion capture data, and joint angle information for each participant and task. Hierarchical data structure of (**b**) raw EMG signal data and (**c**) processed EMG signal data.

The dataset is available at Comprehensive Human Locomotion and Electromyography Dataset: Gait120^46^. All participant data have been anonymized, and no personally identifiable information is included in the repository. The dataset is structured by participant, with individual folders named “S001” to “S120”. Each participant folder contains three main subdirectories: “MotionCapture”, “JointAngle”, and “EMG”. The overall structure of a data record is shown in Fig. 3. For convenience, the dataset is packaged into multiple compressed files, each containing all data from ten subjects. The total dataset size is 15.38 GB, and each zip file is approximately 1.3 GB in size. Table 1 provides an overview of the file organization, including the contents and sizes of each file.

**Table 1:** Overview of the dataset organization.

File Name	File Size	Contents
Gait120_001_to_010.zip	1.35 GB	Subjects 1–10
Gait120_011_to_020.zip	1.32 GB	Subjects 11–20
Gait120_021_to_030.zip	1.28 GB	Subjects 21–30
Gait120_031_to_040.zip	1.28 GB	Subjects 31–40
Gait120_041_to_050.zip	1.26 GB	Subjects 41–50
Gait120_051_to_060.zip	1.25 GB	Subjects 51–60
Gait120_061_to_070.zip	1.33 GB	Subjects 61–70
Gait120_071_to_080.zip	1.25 GB	Subjects 71–80
Gait120_081_to_090.zip	1.28 GB	Subjects 81–90
Gait120_091_to_100.zip	1.32 GB	Subjects 91–100
Gait120_101_to_110.zip	1.16 GB	Subjects 101–110
Gait120_111_to_120.zip	1.28 GB	Subjects 111–120
Total Size	15.38 GB	Data from 120 subjects included

The dataset consists of multiple compressed files, each containing motion capture and electromyography (EMG) data from ten subjects.

### Motion capture data

The “MotionCapture” folder contains eight task-specific folders: “Static”, “LevelWalking”, “StairAscent”, “StairDescent”, “SlopeAscent”, “SlopeDescent”, “SitToStand”, and “StandToSit”. The “Static” folder contains marker trajectory data used for building the Plug-in Gait model. All other task folders contain marker trajectory and sEMG signal data specific to each task. Each task folder includes two subfolders:

1. The “TRC” folder contains marker trajectory data in .trc format, used for inverse kinematics in the OpenSim software. These files were exported from Vicon Nexus, filtered, and segmented into single steps using MATLAB. All marker trajectories were recorded in millimeters.
2. The “MOT” folder contains force plate data in.mot format, used for inverse dynamics in the OpenSim software. These files were exported from Vicon Nexus and segmented into single steps using MATLAB. The force plate data are available only for level walking, sit-to-stand, and stand-to-sit tasks. The force plate data for the first ten participants (S001 to S010) are not available because the force plate coordinate system was not correctly aligned with the motion capture system during the experiment. Force data were recorded in newtons, torque data were recorded in newton-meters (Nm), and moment arms were recorded in meters.

### Joint angle data

**Fig. 4:**
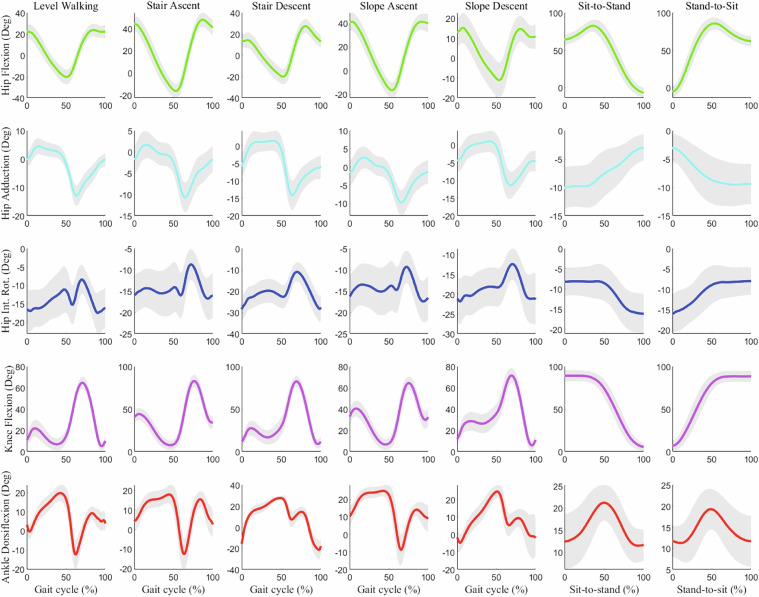
Joint Kinematics of the right lower limb. Mean joint angles (±SD) during seven locomotion tasks for 120 participants. Positive values indicate hip flexion, hip adduction, hip internal rotation, knee extension, and ankle dorsiflexion.

The “JointAngle” folder contains joint angle data calculated using the OpenSim inverse kinematics tool, stored in.mot format (Fig. 4). Joint The folder contains seven task-specific directories: “LevelWalking”, “StairAscent”, “StairDescent”, “SlopeAscent”, “SlopeDescent”, “SitToStand”, and “StandToSit”. Each task folder contains multiple trial folders, and within each trial folder,.mot files are named according to their step index (e.g., “Step01.mot”, “Step02.mot”). Joint angles were calculated and recorded in degrees.

### Surface EMG signal data

The “EMG” folder contains both raw and processed sEMG signal data, stored in MATLAB.mat file format: “RawData.mat” and “ProcessedData.mat”. The “RawData.mat” file (Fig. 3(b)) contains sampling frequencies (sEMG and motion capture), experimental sEMG data, and frame information. Surface EMG signals were recorded in volts. The EMG data are provided in.mat format rather than CSV because of the large file size associated with text-based formats. The provided.mat files can be readily opened and analyzed using GNU Octave, a free, publicly available software platform. Each task-specific field contains two data structures:

1. “MVC_raw”: Contains raw sEMG signals recorded during maximum voluntary contraction (MVC) measurements
2. “Trial00”: Includes raw sEMG signals recorded during the task, frame information for captured signals, number of steps, and “Step00” subfield containing start and end frame information for each step

**Fig. 5:**
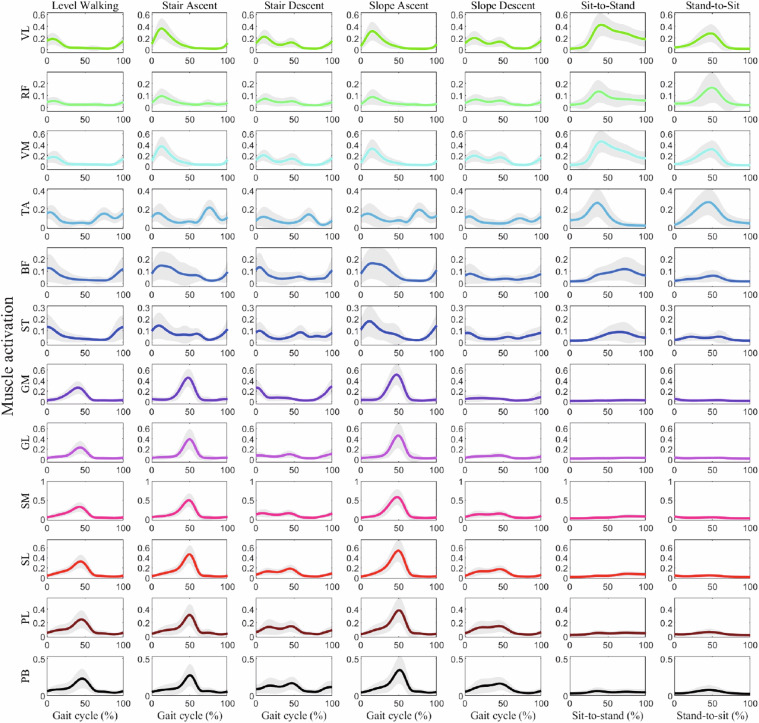
Surface EMG activity of the right lower limb muscles. Mean normalized EMG amplitude (±SD) from 12 muscles during seven locomotion tasks (N = 120). Positive values indicate higher muscle activation relative to maximum voluntary contraction (MVC).

The “ProcessedData.mat” file (Fig. 3(c)) contains filtered and rectified sEMG signals from both MVC measurements and experimental tasks (Fig. 5). The file is organized into seven task-specific fields, each containing enveloped MVC sEMG signals, number of trials, and trial-specific sEMG data. Each trial field includes number of extracted steps, MVCs used for data normalization, and step-specific sEMG data. The step-specific data contains four processing stages:

1. “EMGs_filt”: Filtered sEMG signals.
2. “EMGs_env”: Enveloped signals after filtering
3. “EMGs_norm”: MVC-normalized sEMG data
4. “EMGs_interpolated”: Data interpolated to zero at start and one at end

### Data limitations

This dataset has several limitations. First, data were collected exclusively from healthy adult males aged 20–59, limiting generalizability to females, children, older adults, or clinical populations. Experiments were conducted in a controlled laboratory setting at self-selected speeds, and thus may not fully represent real-world or more dynamic activities. Heel-strike and toe-off events during stair and slope tasks were identified manually, introducing potential subjectivity. Force plate data for the first ten participants are unavailable due to alignment issues. Additionally, surface EMG measures superficial muscle activity and may be influenced by factors such as skin movement or sweating, potentially affecting signal quality and interpretation.

## Technical Validation

### Experimental protocol

One main observer (DS) supervised and validated the placements of markers and sEMG sensors for all experiments to reduce observer variability. Prior to each session, camera calibration was performed following the manufacturer’s standard procedures, including strobe intensity adjustment and threshold value optimization for marker visibility. All digital equipment underwent standard inspection protocols.

If markers or sEMG sensors became detached during testing, the task was paused, and participants were given adequate rest before repeating MVC measurements and the task. Detailed trial information documented any issues encountered during data collection and processing. The main observer performed all marker labelling and gap-filling by using Vicon Nexus software, employing spline and pattern filling with a maximum gap restriction of 20 frames^47^. During the data validation process, trials with missing motion capture markers and abnormal sEMG signals were manually identified and removed from the dataset.

### Reported accuracy of marker-based inverse kinematics

Repeatability of marker-based inverse kinematics has been evaluated in both human-subject and model-based studies. In human-subject experiments, within-subject repeatability has been reported to be high in the sagittal plane, with coefficients of multiple correlation exceeding 0.99^48^. Greater variability has been reported in the frontal and transverse planes. This variability has been associated with differences in marker placement and anatomical landmark identification.

In a separate study, a robotic model was used to evaluate errors under conditions where soft tissue artefacts and anatomical ambiguity were removed^49^. Under these conditions, RMS errors for joint angles across six lower-extremity degrees of freedom ranged from 2.12 ± 1.30° for hip flexion to 8.75 ± 5.05° for ankle inversion. The RMS error for marker reconstruction was 12.9 ± 7.0 mm.

### Comparison of the joint kinematics with public dataset

Although our dataset includes full-body kinematics, we compared our data with a previous study of 10 participants that included lower limb kinematics for all seven tasks^50^. The reference dataset consisted of healthy individuals with similar demographics (mean ± SD age: 30.4 ± 14.9 years) to our study population (35.2 ± 12.9 years).

In our study, level walking was performed at self-selected speeds, with a mean velocity of 1.2 m/s. For validation, we selected a reference dataset with matching walking speed (1.2 m/s) from the public dataset. For stair locomotion, we used stairs with 10 cm step height and compared our results with reference dataset collected on stairs of similar height (9.7 cm). Similarly, for slope walking, we compared our data collected on a 10.1° inclined walkway with a reference dataset obtained at a closely matching incline angle of 10.0°.

Cross-correlation analysis was performed between the reference dataset^50^ and our experimental data for five joint angles (hip flexion/extension, hip abduction/adduction, hip internal/external rotation, knee flexion/extension, and ankle flexion/extension) across all seven tasks, as summarized in Table 2. There were high correlations between the two datasets except for hip internal/external rotation and ankle flexion/extension during the sit-to-stand and stand-to-sit tasks. The joint kinematics of the reference dataset^50^ were calculated using Vicon Nexus software, which defines the hip internal/external rotation angles relative to a static standing posture. In contrast, our dataset utilized a bone-attached coordinate system implemented in OpenSim^6^. These methodological differences resulted in the low and occasionally negative correlation coefficients observed for hip internal/external rotation in Table 2. Additionally, participants in the reference dataset appeared to position their feet closer to the stool than those in our study.

**Table 2:** Cross-correlation coefficients of joint angles between reference dataset^50^ and current study data across seven locomotion tasks.

	Level walking	Stair ascent	Stair descent	Slope ascent	Slope descent	Sit-to-stand	Stand-to-sit
Hip flexion/extension	0.99	0.96	0.80	1.00	0.99	0.88	0.81
Hip abduction/adduction	0.97	0.73	0.92	0.93	0.94	0.97	0.93
Hip internal/external rotation	−0.25	0.39	0.69	0.17	0.60	0.90	0.94
Knee flexion/extension	0.97	0.98	0.94	1.00	0.98	0.95	0.94
Ankle flexion/extension	0.91	0.65	0.95	0.96	0.90	0.31	0.26

## Data Availability

MATLAB code for processing sEMG signal data are provided to demonstrate the sEMG signal processing pipeline and MVC calculation methods. The code “signal_processing.m” filters and rectifies the sEMG signal data. The code “MVC_calculation.m” determines the MVCs for each muscle using sEMG signals from both MVC measurements and experimental tasks. The file ‘data_visualization.m’ is provided for visualizing joint kinematics and sEMG signals.
